# Understanding COVID-19 Vaccination Intention in Japan: The Influence of Individual Social Capital and Personal Beliefs on Vaccination

**DOI:** 10.3390/vaccines14090795

**Published:** 2026-09-09

**Authors:** Yutaro Furukawa, Mikiko Tokiya, Takaomi Kobayashi, Xueyan Zhang, Megumi Hara

**Affiliations:** 1 Department of Preventive Medicine, Faculty of Medicine, Saga University, 5-1-1 Nabeshima, Saga 849-8501, Japan; furukawa@toyu-kai.com (Y.F.); takaomi_920@yahoo.co.jp (T.K.); 2 Department of Social and Environmental Medicine, Faculty of Medicine, Saga University, 5-1-1 Nabeshima, Saga 849-8501, Japan; sx4932@cc.saga-u.ac.jp; 3 School of Public Health, University of Pittsburgh, 130 Desoto St, Pittsburgh, PA 15261, USA; xuz55@pitt.edu

**Keywords:** COVID-19 vaccination intention, 7C model, vaccine hesitancy, personal beliefs on vaccination, individual social capital, public health intervention

## Abstract

**Background/Objectives**: Vaccine hesitancy remains a significant public health challenge in Japan, particularly following the HPV vaccine suspension (2013–2022) that severely damaged vaccination trust. Although prior studies have linked individual social capital to vaccination behavior in Japan, whether and how personal vaccination beliefs modify this association remains poorly understood. This study examined associations between individual social capital (resources accessed through personal social networks) and COVID-19 vaccination intention, and how these associations interact with personal vaccination beliefs based on the 7C model. **Methods**: A nationwide web-based survey (January 2024) included 7210 participants aged 20–69. Individual social capital was measured using civic participation, social cohesion (community trust), and reciprocity (mutual helping). Personal beliefs were assessed via the 7C model (psychological vaccination determinants framework) and factor-analyzed. Logistic regression examined associations; interaction analyses tested whether social capital effects differed across belief patterns. **Results**: Overall, 73.1% expressed intention to receive COVID-19 vaccination for 2024–2025. All social capital components positively associated with vaccination intention: social cohesion (adjusted OR = 1.80, 95% CI:1.58–2.04), reciprocity (adjusted OR = 1.64, 95% CI:1.33–2.02), and civic participation (adjusted OR = 1.29, 95% CI:1.10–1.51). Factor analysis identified three belief domains: individual and collective health responsibility, trust in vaccination systems, and safety concerns. Health responsibility beliefs substantially reduced social capital–vaccination associations when included in models. Safety concerns moderated effects of social cohesion (*p* < 0.001) and reciprocity (*p* = 0.011), with stronger effects among those with lower concerns. Civic participation was associated with vaccination intention mainly among those with higher institutional trust. **Conclusions**: Individual social capital was significantly associated with vaccination intention through complex belief interactions. Safety concerns act as crucial moderators. Multifaceted strategies addressing social capital, safety concerns, and institutional trust may be needed to increase vaccine uptake.

## 1. Introduction

According to the World Health Organization, vaccine hesitancy, reluctance or uncertainty about getting vaccinated, is one of the top 10 threats to global health [1,2]. Importantly, hesitancy is not the same as refusal. Hesitant individuals may decide to get vaccinated despite doubts, or they may delay vaccination; however, many ultimately refuse it [3,4]. Vaccination intention, a person’s plan to get vaccinated, is influenced by hesitancy and by other psychological and contextual factors [5].

Japan has faced unique challenges with vaccine confidence. From 2013 to 2022, the government suspended its active promotion of human papillomavirus (HPV) vaccines after public concerns about side effects, leading to much lower vaccination rates compared to other developed countries [6,7]. This suspension severely damaged public trust in the vaccination system, and a global study found that Japan had the lowest vaccine confidence (trust in safety and effectiveness) among countries surveyed [8].

The COVID-19 pandemic brought new challenges to vaccination efforts worldwide. Before Japan’s vaccination program started in April 2021, researchers estimated that 11.3% to 37.9% of Japanese adults were hesitant about COVID-19 vaccines, similar to the global average [9,10,11]. Despite these initial concerns, by March 2023 Japan achieved remarkably high vaccination coverage, with one of the world’s highest booster vaccination rates [12]. However, as routine boosters became available, maintaining vaccination intention became more difficult [13].

During the pandemic, several factors affected vaccination behavior, including exposure to misinformation, social pressure, and trust in health authorities [14,15]. Studies in Japan found that younger age, female sex, lower income, psychological distress, and worries about side effects were linked to vaccine hesitancy [9,13]. To better understand what drives vaccination decisions, researchers developed the 7C model [14,15]. This model expanded the earlier 5C framework (confidence, complacency, constraints, calculation, collective responsibility) by adding conspiracy beliefs and compliance, providing a comprehensive assessment of beliefs and attitudes that affect vaccination behavior [15].

Social capital, features of social relationships, including interpersonal trust, norms of reciprocity, and civic engagement that facilitate collective action [16,17], has emerged as an important factor in vaccination attitudes [18,19]. Importantly, social capital can be measured at different levels. Community-level social capital (hereafter, community SC) refers to collective characteristics of communities or regions, whereas individual-level social capital (hereafter, individual SC) refers to resources that individuals access through their personal social networks and relationships. This study focuses on individual SC, which encompasses both cognitive components (such as perceived trust and norms of reciprocity) and structural components (such as civic participation and social connections). Unlike community SC, which characterizes entire communities, individual SC captures personal attributes related to one’s engagement with and perception of their social environment [16,17].

Evidence from COVID-19 vaccination studies consistently shows that social capital is associated with vaccine acceptance (positive attitudes toward and uptake of vaccination). In Canada, greater community trust was linked to higher willingness to get COVID-19 vaccines [19]. In Japan, Machida and colleagues found that individual SC (civic participation, social cohesion, and reciprocity) was associated with higher COVID-19 vaccine receipt and booster intention [20]. At the community level, county-level studies in the United States demonstrated that areas with higher community SC had greater vaccination uptake [21,22], and community engagement, a related, but distinct concept, was associated with vaccine uptake in China [23]. While these findings, including in Japan [20], establish associations between social capital and vaccination, whether and how individual SC interacts with psychological factors such as personal beliefs about vaccines—particularly safety concerns and trust in vaccination systems—remains unclear, representing a key gap this study addresses.

Our study had two main goals. First, we examined associations between individual SC factors and COVID-19 vaccination intention. Second, we investigated whether these associations differed depending on people’s underlying beliefs about vaccines, as measured by the 7C model [14,15]. This approach allowed us to understand how social factors and individual attitudes jointly shape vaccination decisions.

## 2. Materials and Methods

### 2.1. Study Sample and Data Collection

This study used data from a nationwide web-based cross-sectional survey conducted between 26 and 29 January 2024. Panel participation was voluntary, and translatable monetary credits, which could be used to buy products and services from corporate sponsors, were given to those who participated in the survey. These credits are commonly used in research panels in Japan and are considered a form of translatable monetary compensation. Our previous studies reported the survey details [10,24]. The survey included men and women aged 20–69, residing in Japan who were registered with Macromill Inc., Tokyo, Japan. Participants were recruited through email registration. We used quota sampling with a predetermined target of 7000 respondents, and data collection continued until this threshold was reached. Therefore, a conventional response rate is not applicable for this study. The survey questionnaire consisted of the Social Capital indicators questionnaire [25], the 7C model questionnaire [15,26]. Vaccination intention was assessed using a similar approach to Nomura et al. [13]. The 7C model questionnaire translated into Japanese, has been assessed in previous studies [27] and the grammatical ambiguity noted in the English translation does not exist in the Japanese version. While a preliminary survey of the entire questionnaire was not conducted, the web survey was designed to prevent respondents from proceeding to the next question unless they correctly answered the current question, thereby avoiding missing responses and contradictory answers. This web survey has already been conducted multiple times using the same questionnaire, and those results have been published in several papers [10,24,27]. The questionnaire was uploaded to a secure section of the website and participants provided information about their COVID-19 vaccination history, their intentions to vaccinate during 2024–2025, and basic sociodemographic information. Survey instructions were sent via email. Answering the questionnaire was considered evidence of consent. This study was approved by the Ethics Committee of Saga University, Saga, Japan (No: R5-28) on 27 December 2023.

### 2.2. Measures

#### 2.2.1. Vaccination Intention for the Next Season

The outcome variable was COVID-19 vaccination intention for the next season (October 2024 to March 2025). Participants rated their agreement with 10 vaccination intention statements on a 5-point Likert scale (from 1 = strongly disagree to 5 = strongly agree):

- I would get vaccinated if it were free.
- I would get vaccinated even if I had to pay.
- I would get vaccinated if everyone else did.
- I would get vaccinated for occupational reasons.
- I would get vaccinated if the effect lasted longer.
- I would get vaccinated if side effects were mild.
- I would get vaccinated if it were developed in Japan.
- I would get vaccinated if I could choose from various types of vaccines according to my preference.
- I would get vaccinated if no appointment was needed.
- I would get vaccinated if it were available near my home or workplace.

Participants who responded with either “agree” or “strongly agree” to any of these statements were categorized as having vaccination intention for the 2024–2025 season. Those who responded with “strongly disagree,” “disagree,” or “neutral” to all statements were categorized as not having vaccination intention. Because agreement with any single conditional statement was sufficient for classification, this measure captures a broad, conditional form of willingness to vaccinate rather than a firm, unconditional intention.

#### 2.2.2. Individual Social Capital

Individual social capital, the explanatory variable in this study, was assessed using three indicators: civic participation, social cohesion, and reciprocity. These measures were developed and validated by the Japan Gerontological Evaluation Study [25]. Civic participation was measured by the frequency of involvement in social groups such as volunteer groups, sports clubs, hobby activity groups, study/cultural groups, or groups focused on teaching and sharing skills and experiences. Participants engaging in any of these groups at least once a month were categorized as having “any participation,” whereas those participating less than once a month were classified as having “no participation.” Social Cohesion was assessed using three questions:

- Do you think people living in your area can be trusted in general?
- Do you think people living in your area often help others?
- What is your level of attachment to the area where you currently live?

Participants were scored as having “social cohesion” if they responded “very much” or “moderately” to at least one of the three questions. Those who answered “neither,” “not much,” or “not at all” to all questions were categorized as having no “social cohesion.” Reciprocity was evaluated using three questions:

- Do you have someone to talk to about your concerns and complaints?
- Do you listen to someone’s concerns and complaints?
- Do you have someone to take care of you when you are sick or in bed for a few days?

“Presence of support” was considered existent if participants responded positively to at least one question, whereas support was considered lacking if participants responded “no one” to all three questions. For each dimension of social capital, the cut-off was chosen with the same method as previous literature to maintain consistency, enabling more direct comparisons with prior findings [20,25].

#### 2.2.3. Personal Beliefs on Vaccination, Including the 7C Model

To assess personal beliefs about vaccination, we prepared 15 survey questions, including items from the 7C model [15] and additional questions to capture a broad range of vaccination attitudes and beliefs. Participants rated their agreement with each statement on a 7-point Likert scale (from 1 = strongly disagree to 7 = strongly agree). Responses were categorized as “disagreed” for scores of 1 (strongly disagree), 2 (disagree), 3 (somewhat disagree), and “agreed” for scores of 5 (somewhat agree), 6 (agree), or 7 (strongly agree). A neutral response (score of 4) was categorized as “neutral” to ensure a more robust analysis, assessing only clear positive and negative responses. For each factor identified through factor analysis, if participants agreed with any question in that factor domain, the factor was categorized as “agreed,” whereas if they disagreed with all questions in that domain, it was categorized as “disagreed.” These statements included:

- I get vaccinated because it is too risky to get infected.
- I only get vaccinated when the benefits clearly outweigh the risks.
- I see vaccination as a collective task against the spread of diseases.
- Vaccination is important for my health.
- Vaccination can reduce the chances of infection for those around the vaccinated person.
- Vaccinations are so important to me that I prioritize getting vaccinated over other things.
- I am convinced that appropriate authorities only allow effective and safe vaccines.
- It should be possible to sanction people who do not follow the vaccination recommendations by health authorities.
- It is easy to obtain accurate information about vaccination.
- I have a correct understanding of the vaccinations I have received so far.
- Vaccinations cause diseases and allergies that are more serious than the diseases they ought to protect from.
- Newly developed vaccines are more dangerous than previously existing vaccines.
- I am concerned about the severe side effects of vaccination.
- Vaccination against diseases that have already become rare in Japan is unnecessary.
- If everyone around me is vaccinated, I do not need to get vaccinated.

#### 2.2.4. Covariates

Participants provided demographic information, including sex, age (categorized as 20–59 or 60–69 years), marital status (married or unmarried), employment status (employed or unemployed), and the presence of children (aged 6 months to 12 years) in the household. Socioeconomic status was assessed based on educational attainment (categorized as university graduate or university graduate and above). Health status was evaluated based on the presence of underlying diseases. Participants were classified as having comorbidities based on self-reported conditions including diabetes, hypertension, hyperlipidemia, heart disease, chronic kidney disease, asthma and chronic respiratory disease, immune deficiency, allergic disease, and liver disease. For analysis, we categorized participants into three groups: those with no comorbidities, those with one comorbidity, and those with two or more comorbidities. Household income was evaluated by categorizing participants into three groups based on annual household earnings: low income (less than 4,000,000 yen, approximately $27,000 USD), middle income (4,000,000 to 8,000,000 yen, approximately $27,000–$54,000 USD), and high income (greater than 8,000,000 yen, approximately $54,000+ USD).

### 2.3. Statistical Analysis

We employed multiple analytical approaches to examine relationships among social capital, personal beliefs, and vaccination intentions. First, bivariate analyses were conducted to examine associations between sociodemographic characteristics, social capital components, and vaccination intention using chi-squared tests. We then analyzed associations between individual personal beliefs and vaccination intentions using logistic regression to calculate crude odds ratios (ORs) with 95% confidence intervals (CIs). To address potential multicollinearity among the 15 personal belief items, we conducted an exploratory factor analysis with a promax rotation. The optimal number of factors was determined using a scree plot. Items with factor loadings greater than 0.4 were retained, and in cases where items loaded substantially on multiple factors, they were assigned to the factor with the strong loading and conceptual fit. Reliability of identified factors was assessed using Cronbach’s alpha, with values above 0.70 considered acceptable.

We then conducted a series of logistic regression analyses to examine associations between identified personal belief factors and vaccination intention by calculating both crude and adjusted odds ratios. Adjustments were made for sociodemographic variables including sex, age, educational attainment, presence of underlying diseases, marital status, household income, employment status and presence of children under 12 years of age. Finally, to examine the relationship between individual social capital and vaccination intention, multiple logistic regression analyses were performed using a stepwise approach. Model 1 examined crude associations, Model 2 adjusted for sociodemographic factors, and Models 3–5 additionally adjusted for each personal belief factor separately to examine their moderating effects on the relationship between social capital and vaccination intention. Interactions between social capital components and personal belief factors were assessed through logistic regression analysis using interaction terms. All statistical analyses were performed using SAS version 9.4, with statistical significance set at *p* < 0.05.

## 3. Results

### 3.1. Sociodemographic Characteristics and COVID-19 Vaccination Intentions

Our study included 7210 participants, with 73.1% expressing intention to receive the COVID-19 vaccination for 2024–2025 (Table 1). All social capital components were positively associated with vaccination intention: participants with civic participation (79.3% vs. 71.5%, *p* < 0.0001), social cohesion (78.4% vs. 64.2%, *p* < 0.0001), and reciprocity (74.4% vs. 60.5%, *p* < 0.0001) showed higher vaccination intention. Demographic factors associated with higher vaccination intention included: male sex (74.8% vs. 71.7%, *p* = 0.0036), older age (80.8% vs. 69.3%, *p* < 0.0001), university education (76.7% vs. 70.2%, *p* < 0.0001), having underlying diseases (84.7% vs. 69.5%, *p* < 0.0001), being unmarried (75.7% vs. 69.3%, *p* < 0.0001), and being unemployed (77.2% vs. 72.5%, *p* = 0.0016). Those living with children under 12 showed lower vaccination intention (69.1% vs. 73.9%, *p* = 0.0008). Household income differences were not statistically significant. Multivariate analysis of covariates revealed higher education, having underlying diseases, being unmarried, and not living with young children are positively related to vaccination intention after adjustments (Table S4).

### 3.2. Analysis of Personal Beliefs on Vaccination

Analysis of individual personal beliefs showed varying associations with vaccination intentions in Table S1. The strongest positive associations with vaccination intention were observed for belief items related to vaccination prioritization (OR = 24.86, 95% CI: 19.09–32.38), infection risk concerns (OR = 24.29, 95% CI: 20.02–29.47), and importance for personal health (OR = 19.21, 95% CI: 16.23–22.74), followed by trust in authorities (OR = 11.04, 95% CI: 9.32–13.08) and vaccination reducing infection for others (OR = 10.43, 95% CI: 9.02–12.08). Conversely, concerns about vaccines causing serious conditions (OR = 0.37, 95% CI: 0.33–0.43) and beliefs that newly developed vaccines are more dangerous (OR = 0.45, 95% CI: 0.39–0.53) showed negative associations with vaccination intention. While all 15 personal belief items were significantly related to vaccination intention, analyzing them separately would be problematic because of their interconnected nature. Having too many correlated variables makes it difficult to interpret results and reduces the statistical power of the analysis. To solve this problem, we use factor analysis to identify the underlying belief patterns and conduct a more robust analysis.

Factor analysis of the 15 personal belief items related to vaccination identified three distinct domains that together explained 48.2% of the total variance across the 15 items (Table 2). Based on the scree plot (Figure S1), a three-factor structure was deemed optimal: (1) individual and collective health responsibility (Factor 1), (2) trust in vaccination systems and information (Factor 2), and (3) vaccine safety concerns (Factor 3). Of the common variance extracted by these three factors, Factor 1 accounted for 39.4% (Cronbach’s α = 0.860), Factor 2 for 31.1% (Cronbach’s α = 0.753), and Factor 3 for 29.4% (Cronbach’s α = 0.749). Four items showed substantial loadings (>0.4) on both Factors 1 and 2: “I get vaccinated because it is too risky to get infected” (item 1 from Table 2), “Vaccination is important for my health” (item 4 from Table 2), “Vaccinations are so important to me that I prioritize getting vaccinated over other things” (item 6 from Table 2), and “I am convinced the appropriate authorities only allow effective and safe vaccines” (item 7 from Table 2). These items were assigned to factors that showed strong loadings, and their conceptual fit within their respective domains was confirmed.

Personal beliefs about vaccination were strongly associated with vaccination intentions (Table 3). Individual and collective health responsibility (Factor 1) demonstrated the strongest positive association, followed by trust in vaccination systems and information (Factor 2). By contrast, vaccine safety concerns (Factor 3) were negatively associated with vaccination intention. These associations remained consistent in direction and significance after adjusting for sociodemographic factors, although their magnitude was slightly attenuated.

### 3.3. Association Between Individual Social Capital and COVID-19 Vaccination Intention

Logistic regression analyses revealed significant associations between social capital factors and vaccination intentions (Table 4). All three components of individual social capital showed positive associations with vaccination intention in both crude and adjusted analyses for sociodemographic factors. Associations between social capital components and vaccination intention were affected differently by adjustments for personal belief factors. For social cohesion, the strongest reduction in association was observed after adjusting for individual and collective responsibility for health, followed by a moderate reduction in trust in vaccination systems, whereas concerns about vaccine safety had minimal impact. A similar pattern was observed for reciprocity. Among all social capital components, the adjustment for concerns about vaccine safety consistently showed the smallest reduction.

### 3.4. Interactions of Personal Beliefs and Individual Social Capital on COVID-19 Vaccination Intention

The relationship of social capital with vaccination intention varies meaningfully across belief contexts (Table 5). For vaccine safety concerns, we found strong interactions with social cohesion (*p* < 0.0001) and reciprocity (*p* = 0.011). The association between social cohesion and vaccination intention was dramatically stronger among those with low safety concerns (OR = 4.04) compared to those with high concerns (OR = 1.47), suggesting that the positive association between social capital and vaccination intention was weaker as safety concerns increased. For trust in vaccination systems, we found a significant interaction with civic participation (*p* = 0.004). Civic participation was associated with higher vaccination intention only among those with high system trust (OR = 1.52), with no association observed among those with low trust (OR = 0.88). This suggests that the association between civic participation and vaccination intention may depend on institutional trust. No significant interaction was observed between trust in vaccination systems and social cohesion (*p* = 0.447). For individual and collective responsibility on health, we did not find any interactions with social capital components. This suggests that health responsibility functions differently than other beliefs, operating as an independent correlate of vaccination intention rather than primarily moderating the association with social capital.

## 4. Discussion

Our study demonstrated that all individual social capital components were significantly associated with individual intentions to receive COVID-19 vaccinations in Japan. Social cohesion showed the strongest relationship, followed by reciprocity and civic participation. Our analysis suggests a significant association between social capital and vaccination intention. Personal beliefs appear to explain part of this relationship, and vaccine safety concerns substantially moderate this association.

Personal beliefs play distinct roles in this relationship. Individual and collective health responsibility demonstrated the most substantial attenuating effect, particularly for social cohesion and reciprocity. Including this factor in our models notably decreased the association between social capital components and vaccination intention. This result suggests that adjustment for individual and collective health responsibility beliefs attenuated the association between social capital and vaccination decisions.

We found significant interactions between trust in vaccination systems and social capital. Civic participation was associated with higher vaccination intention only among individuals with high institutional trust, with no association observed among those with low trust. This pattern suggests that the association between civic engagement and vaccination intention may depend on confidence in health institutions. Conversely, social cohesion showed positive associations regardless of trust levels, suggesting that community cohesion may be associated with vaccination intention even amid institutional distrust, potentially through community-based information sharing [28].

Vaccine safety concerns strongly moderated the association between social capital and vaccination intention. Social cohesion and reciprocity showed substantially stronger positive associations with vaccination intention among individuals with low safety concerns than those with high concerns. These findings indicate that the relationship of social capital with vaccination attitudes is complex and context-dependent [29]. Safety concerns appear to be a powerful moderator that can diminish the otherwise positive associations of social cohesion and reciprocity with vaccination intention. This finding suggests that future intervention research may benefit from addressing safety concerns before implementing social-capital-based strategies to promote vaccination [12,30,31].

While our findings align with previous research linking individual SC to vaccination intention [20], we observed weaker associations for civic participation than reported in earlier studies in China and Japan [20,23]. This difference may reflect pandemic-related reductions in social gatherings, potentially weakening the traditional role of civic participation in health decision-making [21].

Building on these associations, future intervention research could explore tailoring public health strategies to different population segments according to their social capital profiles and belief contexts. For individuals with high social cohesion and reciprocity, messaging that emphasizes individual and collective health responsibility, mutual protection, and community care may be worth evaluating, given that social capital has been linked to altruism [22] and vaccination contributes to community-wide protection. Potential strategies that could be tested in future studies include: (1) community-based participatory research in which residents help design local vaccination campaigns; (2) training respected community members as “health ambassadors” who can discuss vaccination benefits with peers; (3) organizing neighborhood vaccination events that create social momentum and visibility; (4) developing family-centered vaccination initiatives that emphasize protection of vulnerable family members. For civic participation, our findings suggest that without sufficient system trust, even high civic participation may not be associated with higher vaccination intention; future research could examine whether pairing civic-participation strategies with trust-building initiatives, such as creating opportunities for dialogue between health authorities and community members, improves their effectiveness. Given that safety concerns were associated with a diminished positive association between social capital and vaccination intention regardless of community strength, future intervention research should prioritize evaluating whether addressing vaccine safety concerns before implementing social-capital-based strategies improves their effectiveness.

Supporting evidence comes from recent UNICEF studies in which community-based approaches have contributed to increased vaccination rates in various countries [32]. These programs utilize strategies such as mobile vaccination clinics, door-to-door visits by health workers, and community meetings, demonstrating the potential effectiveness of delivering health messages through trusted local channels and addressing community-specific concerns. The success of these programs shows that vaccination efforts work best when health messages are sent through trusted local channels and when interventions are tailored to address community-specific concerns.

Our findings are specific to COVID-19 vaccination in Japan and may not generalize to other vaccines and diseases with different risk profiles. To address this limitation, we examined relationships between social capital dimensions and other vaccination behaviors (Tables S2 and S3). Due to sample-size limitations, we focused on influenza, pneumococcal, and previous COVID-19 vaccination. Results showed patterns resembling our main analysis, with positive relationships between social capital components and vaccination status across different vaccine types. The association between social capital and vaccination intention we observed for COVID-19 is supported by similar findings for other vaccines. Our analysis revealed positive associations between all social capital components, pneumococcal vaccination, and influenza vaccination [33,34]. These consistent patterns across multiple vaccine types suggest that common social mechanisms may be associated with vaccination decisions broadly, though we maintain appropriate caution about generalizing our findings beyond the unique sociopolitical context of COVID-19 vaccination in Japan.

Our study had several limitations that should be considered when interpreting these results. First, the cross-sectional design restricted our ability to establish definitive causal relationships between individual SC and vaccination intention and whether certain personal beliefs mediate their relationship. Second, although our analysis suggested an association between social capital components and vaccination intention, these relationships may operate in either direction. For instance, individuals with reduced vaccination intentions might participate less in community activities or report weaker social cohesion, rather than social capital being the underlying cause of their vaccination decisions. Additionally, our outcome measure classified participants as having vaccination intention if they agreed with any one of ten conditional statements (e.g., willingness to vaccinate if free, if no appointment were needed, or if available nearby); this operationalization may capture conditional willingness rather than a firm intention to be vaccinated, which likely contributed to the relatively high observed prevalence of intention (73.1%). Findings based on this measure should therefore be interpreted as reflecting a broad, conditional form of willingness rather than a strict commitment to vaccinate, and future studies using a stricter, unconditional definition of intention would help clarify how sensitive these associations are to the outcome definition. Third, this study’s generalizability was restricted due to using data from a private research company (Macromill, Inc.) rather than national survey data. This sample was not randomly selected and included only individuals aged 20 to 69, which may not fully represent the general population in Japan. We compared our sample demographics with national data to address this limitation [35], and the results suggest broad similarity. The proportion of female participants in our study was 53.5% compared to 51.3% nationally, and participants aged 60 and older constituted 29.9% of our sample versus 35.4% nationally. The regional distribution was also comparable, with Tokyo comprising 14% of our sample versus 11.1% nationally, and Osaka representing 9.2% versus 7.0% nationally. Despite this demographic balance, self-selection bias remains an issue, particularly regarding attitudes and beliefs about vaccination. Our COVID-19 vaccination intention rate (73.1% among 7210 participants) closely matches that of another Japanese study (72.3% among 2313 participants) [20]. Fourth, at the time of our survey, COVID-19 vaccines were provided free of charge in Japan, and details about the 2024–25 vaccination program had not yet been announced. This timing likely minimized cost considerations relative to stated vaccination intentions of participants. However, it is worth noting that the current 2024–25 program now offers publicly funded vaccines only to those aged 65 or older and individuals 60–64 years with severe underlying conditions, with all others required to pay out of pocket [36]. Anticipating potential financial barriers, we included household income in our analysis. While households earning below 4,000,000 yen annually showed slightly lower odds of vaccination intention compared to higher-income brackets, these differences were not statistically significant (Table S4). This suggests that while financial considerations may contribute to some vaccination decisions, other factors examined in our study appeared more influential. Fifth, social capital levels in Japan including social norms, trust, and civic participation tend to increase with age, with the highest levels typically observed among older adults. This age-related pattern may have led to an underestimation of social capital factors in our study [37]. Additionally, our geographic data were limited to prefectures rather than neighborhoods, preventing community-level social capital assessment. Previous research has established that meaningful social capital analysis in Japan requires data from smaller administrative units, limiting our ability to evaluate social capital in its broader sense [25]. Finally, the reliance on self-reported data may have introduced recall and social desirability biases, potentially affecting accuracy of our findings. This is particularly relevant in the Japanese context, where collective responsibility is strongly associated with social behavior and decision-making, as demonstrated by the stronger association of collective responsibility on vaccination intentions among Japanese and Japan-born participants compared to foreign-born immigrants [38]. Given this cultural emphasis on collective responsibility, participants might have overstated both their social capital and their vaccination intentions. This potential overestimation could have inflated observed associations between individual SC and vaccination intentions in our study.

Future research should include a wide age range of participants with geographic information at the neighborhood level. They should also employ longitudinal study designs to better establish causal relationships, and investigate the interplay between individual SC, personal beliefs, and vaccination intention for COVID-19. Given that our exploratory findings for pneumococcal and influenza vaccination showed similar patterns, future studies could extend this framework beyond COVID-19 to other adult immunizations, such as seasonal influenza and respiratory syncytial virus (RSV) vaccination, as well as to childhood immunization and the caregiver decision-making processes that shape it. Because our study assessed self-reported intention rather than actual vaccination behavior, future research linking survey responses to administrative or registry-based vaccination records would help clarify how closely stated intention aligns with actual COVID-19 vaccination coverage in this population. Social capital itself is shaped by political and social context, including national leadership and the broader political climate, and these contexts vary substantially across countries and over time. While the general mechanism by which individual social capital operates through personal beliefs to shape vaccination intention may be common across settings, whether the specific pattern of associations observed in this study—for example, which beliefs moderate this relationship most strongly—would replicate in other countries remains unknown. Comparative studies across different national and political contexts would be needed to clarify the extent to which these findings generalize beyond Japan.

## 5. Conclusions

Our study revealed significant associations between individual SC components and COVID-19 vaccination intention in Japan for 2024–2025. Social cohesion was the strongest predictor, followed by reciprocity and civic participation. Our findings suggest that adjustment for beliefs concerning individual and collective health responsibility attenuated the association between social capital and vaccination decisions. At the same time, vaccine safety concerns significantly moderated these relationships. These results underscore the potential value of future public health strategies that account for social capital alongside specific beliefs and concerns to increase vaccine uptake.

## Figures and Tables

**Table 1:** Association between explanatory variables and Intention of COVID-19 vaccination.

			Intention of COVID-19 Vaccination				
		Total	No		Yes		
Characteristics		n	n	%	n	%	*p*-Value
Total		7210	1937	26.9	5273	73.1	
Civic participation	No participation	5705	1626	28.5	4079	71.5	<0.0001
	Any participation	1505	311	20.7	1194	79.3	
Social cohesion	No social cohesion	2675	958	35.8	1717	64.2	<0.0001
	Any social cohesion	4535	979	21.6	3556	78.4	
Reciprocity	No reciprocity	658	260	39.5	398	60.5	<0.0001
	Any reciprocity	6552	1677	25.6	4875	74.4	
Sex	Men	3356	847	25.2	2509	74.8	0.0036
	Women	3854	1090	28.3	2764	71.7	
Age	20–59 years old	4817	1477	30.7	3340	69.3	<0.0001
	60–69 years old	2393	460	19.2	1933	80.8	
Educational level	Below university graduate	3957	1179	29.8	2778	70.2	<0.0001
	University graduate or above	3253	758	23.3	2495	76.7	
Underlying disease	0	4874	1486	30.5	3388	69.5	<0.0001
	1	1625	342	21.1	1283	79.0	
	≥2	710	109	15.4	601	84.7	
Marriage Status	Unmarried	4353	1060	24.3	3293	75.7	<0.0001
	Married	2857	877	30.7	1980	69.3	
Job employment	Unemployed	993	226	22.8	767	77.2	0.0016
	Employed	6217	1711	27.5	4506	72.5	
Living with children under 12 years old	No	6100	1594	26.1	4506	73.9	0.0008
	Yes	1110	343	30.9	767	69.1	
Household income *	−4,000,000	1977	533	27.0	1444	73.0	0.15
	4,000,000–8,000,000	2390	607	25.4	1783	74.6	
	8,000,000−	1205	288	23.9	917	76.1	

* Household Income in Yen.

**Table 2:** Factor loadings and internal reliability of Survey Items for Personal Beliefs.

	Factor 1	Factor 2	Factor 3
1. I get vaccinated because it is too risky to get infected	0.657	0.520	
2. I only get vaccinated when the benefits clearly outweigh the risks	0.637		
3. I see vaccination as a collective task against the spread of diseases	0.791		
4. Vaccination is important for my health	0.709	0.462	
5. Vaccination can reduce the chances of infection for those around the vaccinated person	0.751		
6. Vaccinations are so important to me that I prioritize getting vaccinated over other things	0.566	0.597	
7. I am convinced the appropriate authorities do only allow effective and safe vaccines	0.474	0.561	
8. It should be possible to sanction people who don’t follow the vaccination recommendations by health authorities		0.617	
9. It is easy to obtain accurate information about vaccination		0.713	
10. I have a correct understanding of the vaccinations I have received so far		0.566	
11. Vaccinations cause diseases and allergies that are more serious than the disease they ought to protect from			0.765
12. Newly developed vaccines are more dangerous than previously existing vaccines			0.764
13. I am concerned about the severe side effects of vaccination			0.752
14. Vaccination against diseases that have already become rare in Japan is unnecessary			0.574
15. If everyone around me is vaccinated, I don’t need to get vaccinated			0.555
Relative Proportion of Variance explained	0.394	0.311	0.294
Number of Survey Items	5	5	5
Cronbach’s alpha *	0.860	0.753	0.749

* Cronbach’s alpha > 0.7.

**Table 3:** Association between personal beliefs and Intention of COVID-19 vaccination.

Personal Beliefs on Vaccination	Intention to Vaccinate in 2024–2025			
	Total *n*	With Intention *n*, (%)	Crude OR	Adjusted OR *
Factor 1 (Individual and collective responsibility on health)				
Disagree	451	92 (20.4)	1.00 (ref)	1.00
Neutral	1153	542 (47.0)	**3.46 (2.68, 4.47)**	**4.00 (2.97, 5.40)**
Agree	5606	4639 (82.8)	**18.71 (14.73, 23.77)**	**19.22 (14.55, 25.39)**
Factor 2 (Trust in vaccination system and information)				
Disagree	614	250 (40.7)	1.00 (ref)	1.00
Neutral	2080	1202 (57.8)	**1.99 (1.66, 2.39)**	**2.02 (1.62, 2.51)**
Agree	4516	3821 (84.6)	**8.01 (6.69, 9.56)**	**7.14 (5.77, 8.83)**
Factor 3 (Concerns for vaccine safety)				
Disagree	545	413 (75.8)	1.00 (ref)	1.00
Neutral	1904	1464 (76.9)	1.06 (0.85, 1.33)	1.12 (0.86, 1.46)
Agree	4761	3396 (71.3)	**0.80 (0.65, 0.98)**	0.79 (0.62, 1.01)

OR: odds ratio, ref: reference, Bold: statistically significant with *p* < 0.05. * Adjusted for sex, age, education, comorbidity, marriage status, employment, living with children, income.

**Table 4:** Effect of Personal Beliefs on the association between Social Capital and Vaccination Intention.

Individual-Level Social Capital		Model 1	Model 2	Model 3	Model 4	Model 5
Civic Participation	None	(ref)	(ref)	(ref)	(ref)	(ref)
	Any	OR **1.53**	OR **1.29**	OR **1.27**	OR **1.20**	OR **1.30**
		**(1.33, 1.76)**	**(1.10, 1.51)**	**(1.06, 1.51)**	**(1.01, 1.42)**	**(1.11, 1.52)**
Social Cohesion	None	(ref)	(ref)	(ref)	(ref)	(ref)
	Any	OR **2.03**	OR **1.80**	OR **1.42**	OR **1.52**	OR **1.82**
		**(1.82, 2.25)**	**(1.58, 2.04)**	**(1.23, 1.63)**	**(1.32, 1.73)**	**(1.60, 2.07)**
Reciprocity	None	(ref)	(ref)	(ref)	(ref)	(ref)
	Any	OR **1.90**	**OR 1.64**	OR 1.22	OR **1.45**	OR **1.69**
		**(1.61, 2.24)**	**(1.33, 2.02)**	(0.97, 1.54)	**(1.16, 1.80)**	**(1.37, 2.08)**

OR: odds ratio, ref: reference, Bold: statistically significant with *p* < 0.05. Model 1: unadjusted. Model 2: Adjusted for sex, age, education, comorbidity, marriage, job, living with children, income. Model 3: Adjusted for sex, age, education, comorbidity, marriage, job, living with children, income, and Factor 1. Model 4: Adjusted for sex, age, education, comorbidity, marriage, job, living with children, income, and Factor 2. Model 5: Adjusted for sex, age, education, comorbidity, marriage, job, living with children, income, and Factor 3.

**Table 5:** Interaction of Personal Beliefs and Individual-level Social Capital on Vaccination Intention.

Individual-Level Social Capital	Personal Beliefs		Adjusted OR	*p*-Value for Interaction
Individual and collective responsibility on health (Factor 1)				
Civic Participation	Any (vs. None)	Disagree	1.49 (0.79, 2.81)	0.114
		Neutral	0.89 (0.61, 1.30)	
		Agree	**1.38 (1.12, 1.70)**	
Social Cohesion	Any (vs. None)	Disagree	1.68 (0.98, 2.88)	0.079
		Neutral	1.08 (0.82, 1.42)	
		Agree	**1.54 (1.30, 1.82)**	
Reciprocity	Any (vs. None)	Disagree	1.17 (0.53, 2.55)	0.475
		Neutral	1.03 (0.72, 1.48)	
		Agree	**1.37 (1.02, 1.85)**	
Trust in vaccination system and information (Factor 2)				
Civic Participation	Any (vs. None)	Disagree	0.88 (0.53, 1.46)	**0.004**
		Neutral	0.85 (0.63, 1.13)	
		Agree	**1.52 (1.20, 1.92)**	
Social Cohesion	Any (vs. None)	Disagree	**1.58 (1.07, 2.32)**	0.447
		Neutral	**1.33 (1.08, 1.65)**	
		Agree	**1.59 (1.31, 1.93)**	
Reciprocity	Any (vs. None)	Disagree	1.15 (0.63, 2.11)	0.720
		Neutral	**1.39 (1.02, 1.89)**	
		Agree	**1.52 (1.09, 2.11)**	
Concerns for vaccine safety (Factor 3)				
Civic Participation	Any (vs. None)	Disagree	**1.28 (0.72, 2.28)**	0.610
		Neutral	**1.53 (1.07, 2.19)**	
		Agree	**1.25 (1.03, 1.51)**	
Social Cohesion	Any (vs. None)	Disagree	**4.04 (2.50, 6.55)**	**<0.0001**
		Neutral	**2.90 (2.22, 3.78)**	
		Agree	**1.47 (1.26, 1.72)**	
Reciprocity	Any (vs. None)	Disagree	**2.86 (1.51, 5.43)**	**0.011**
		Neutral	**2.29 (1.62, 3.25)**	
		Agree	**1.33 (1.02, 1.74)**	

OR: odds ratio, Bold: statistically significant with *p* < 0.05. Adjusted for sex, age, education, comorbidity, marriage, job, living with children, income.

## Data Availability

Data presented in this study are available on request from the corresponding author.

## Supplementary Materials

The following supporting information can be downloaded at: https://www.mdpi.com/article/10.3390/vaccines14090795/s1, Figure S1: A scree plot of personal beliefs on COVID-19 vaccination; Table S1: Associations of 15 personal beliefs on vaccination and intention of COVID-19 vaccination in the 2024–2025 season; Table S2: Association of past vaccination history of other vaccines and intention of COVID-19 vaccination in the 2024–2025 season; Table S3: Effect of personal beliefs on the association between individual social capital and past vaccination behavior; Table S4: Multivariate analysis for explanatory variables and intention of COVID-19 vaccination.
