# Supplementary material for: Understanding COVID-19 Vaccination Intention in Japan: The Influence of Individual Social Capital and Personal Beliefs on Vaccination
Source: Vaccines (Basel). 2026 Sep 9;14(9):795. doi: 10.3390/vaccines14090795 (PMC13611750; doi:10.3390/vaccines14090795)
Supplement: Supplementary file 1 [file vaccines-14-00795-s001.zip › vaccines-4519986-supplementary.pdf]

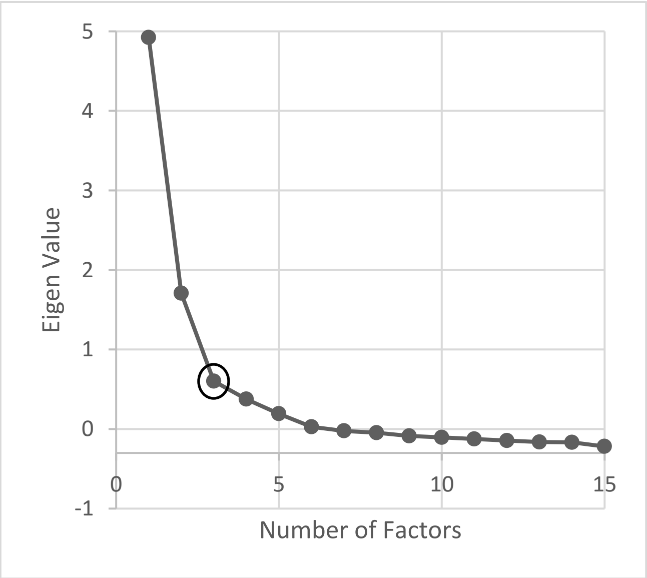

Figure S1. A scree plot of personal beliefs on COVID-19 vaccination

This is a scree plot of personal beliefs on vaccination. The horizontal axis is labeled as the number of factors and the vertical axis is labeled as Eigen value of the 15 questions. The circled point indicates the most appropriate number of factors to retain.

Table.S1 Association of 15 Personal Beliefs on vaccination and Intention of COVID-19 Vaccination in 2024-2025 season

Questions on Personal Beliefs on COVID-19 vaccination	Answer	n	Intention of Vaccination: With Intention *n*, (%)	OR	95% CI
1. I am convinced the appropriate authorities do only allow effective and safe vaccines (confidence)	D	2,376	1,272 (53.5)	1.00	ref
	A	2,566	2,379 (92.7)	**11.04**	**9.32, 13.08**
2. I get vaccinated because it is too risky to get infected (confidence)	D	2,331	1,085 (46.6)	1.00	ref
	A	2,903	2772 (95.5)	**24.29**	**20.02, 29.47**
3. Vaccinations are so important to me that I prioritize getting vaccinated over other things (constraints)	D	3,254	1,802 (55.4)	1.00	ref
	A	1,943	1,882 (96.9)	**24.86**	**19.09, 32.38**
4. I only get vaccinated when the benefits clearly outweigh the risks (calculation)	D	1,764	982 (55.7)	1.00	ref
	A	3,158	2647 (83.8)	**4.13**	**3.61, 4.71**
5. I see vaccination as a collective task against the spread of diseases (collective responsibility)	D	1,345	602 (44.8)	1.00	ref
	A	4,018	3,502 (87.2)	**8,38**	**7.27, 9.65**
6. It should be possible to sanction people who don't follow the vaccination recommendations by health authorities (compliance)	D	4,927	3,388 (68.8)	1.00	ref
	A	651	614 (94.3)	**7.54**	**5.38, 10.56**
7. Vaccinations cause diseases and allergies that are more serious than the disease they ought to protect from (conspiracy)	D	2,430	1,975 (81.3)	1.00	ref
	A	1,915	1183 (61.8)	**0.37**	**0.33, 0.43**
8. Vaccination is important for my health	D	1,806	770 (42.6)	1.00	ref
	A	3,178	2,970 (93.5)	**19.21**	**16.23, 22.74**
9. Vaccination can reduce the chances of infection for those around the vaccinated person	D	1,309	555 (42.4)	1.00	ref
	A	4,037	3,572 (88.5)	**10.43**	**9.02, 12.08**
10. Newly developed vaccines are more dangerous than previously existing vaccines	D	2,100	1,692 (80.6)	1.00	ref
	A	1,530	999 (65.3)	**0.45**	**0.39, 0.53**
11. I am concerned about the severe side effects of vaccination	D	1,766	1,431 (81.0)	1.00	ref
	A	3,732	2,602 (69.7)	**0.54**	**0.47, 0.62**
12. Vaccination against diseases that have already become rare in Japan is unnecessary	D	2,838	2,331 (82.1)	1.00	ref
	A	1,480	1,002 (67.7)	**0.46**	**0.39, 0.53**
13. If everyone around me is vaccinated, I don't need to get vaccinated	D	4,328	3,538 (81.8)	1.00	ref
	A	762	508 (66.7)	**0.45**	**0.38, 0.53**
14. It is easy to obtain accurate information about vaccination	D	2,393	1,534 (64.1)	1.00	ref
	A	1,944	1,665 (85.7)	**3.34**	**2.87, 3.89**
15. I have a correct understanding of the vaccinations I have received so far	D	2,182	1,365 (62.6)	1.00	ref
	A	2,787	2,352 (84.4)	**3.24**	**2.83, 3.70**

OR: odds ratio, CI: confidence interval, ref: reference, Bold: statistically significant with $p<0.05$

D: Disagreed A: Agreed

Table.S2 Association of Past Vaccination History of Other Vaccines and Intention of COVID-19 Vaccination in 2024-25 season

			Intention of Vaccination		
**Past Vaccination History of Other Vaccines**	Answer	n	With Intention *n*, (%)	*OR	95% CI
Influenza Vaccine	No	1,536	931 (60.6)	1.00	ref
	Yes	5,548	4,283 (77.2)	**2.41**	**2.13, 2.73**
Pneumococcal Vaccine	No	4,846	3,463 (71.5)	1.00	ref
	Yes	1,426	1,208 (84.7)	**1.91**	**1.62, 2.25**
COVID-19 Vaccine	No	1,073	397 (37.0)	1.00	ref
	Yes	6,137	4876 (79.5)	**6.31**	**5.49, 7.25**

*OR: adjusted with sex and age

Table S3. Effect of Personal Beliefs on the association between Individual-level Social Capital and Past Vaccination Behavior

Individual-level Social Capital		Model 1	Model 2	Model 3	Model 4	Model 5
**Influenza Vaccine (previously vaccinated)**						
Civic Participation	Any (vs None)	OR **1.24**	OR **1.16**	OR **1.15**	OR **1.14**	OR **1.17**
		**(1.07, 1.43)**	**(0.99, 1.37)**	**(0.97, 1.35)**	**(0.96, 1.34)**	**(0.99, 1.38)**
Social Cohesion	Any (vs None)	OR **1.38**	OR **1.37**	OR **1.25**	OR **1.29**	OR **1.37**
		**(1.23, 1.55)**	**(1.20, 1.57)**	**(1.08, 1.43)**	**(1.13, 1.48)**	**(1.20, 1.57)**
Reciprocity	Any (vs None)	OR **2.18**	OR **1.58**	OR **1.43**	OR **1.51**	OR **1.59**
		**(1.82, 2.60)**	**(1.26, 1.97)**	**(1.15, 1.80)**	**(1.21, 1.88)**	**(1.27, 1.99)**
**Pneumococcal Vaccine (previously vaccinated)**						
Civic Participation	Any (vs None)	OR **2.44**	OR **1.83**	OR **1.81**	OR **1.79**	OR **1.83**
		**(2.15, 2.79)**	**(1.56, 2.15)**	**(1.54, 2.13)**	**(1.52, 2.10)**	**(1.56, 2.15)**
Social Cohesion	Any (vs None)	OR **2.01**	OR **1.52**	OR **1.43**	OR **1.43**	OR **1.51**
		**(1.77, 2.28)**	**(1.30, 1.77)**	**(1.22, 1.68)**	**(1.22, 1.68)**	**(1.29, 1.77)**
Reciprocity	Any (vs None)	OR **2.93**	OR **1.60**	OR **1.48**	OR **1.50**	OR **1.60**
		**(2.17, 3.95)**	**(1.10, 2.32)**	**(1.02, 2.15)**	**(1.04, 2.18)**	**(1.10, 2.32)**
**COVID-19 Vaccine (previously vaccinated)**						
Civic Participation	Any (vs None)	OR **1.67**	OR **1.43**	OR **1.41**	OR **1.36**	OR **1.45**
		**(1.39, 2.00)**	**(1.16, 1.77)**	**(1.13, 1.76)**	**(1.10, 1.69)**	**(1.18, 1.80)**
Social Cohesion	Any (vs None)	OR **1.85**	OR **1.58**	OR **1.23**	OR **1.39**	OR **1.61**
		**(1.62, 2.11)**	**(1.35, 1.85)**	**(1.04, 1.46)**	**(1.18, 1.64)**	**(1.38, 1.89)**
Reciprocity	Any (vs None)	OR **2.16**	OR **1.74**	OR **1.39**	OR **1.59**	OR **1.84**
		**(1.79, 2.61)**	**(1.37, 2.19)**	**(1.08, 1.78)**	**(1.25, 2.02)**	**(1.45, 2.33)**

OR: odds ratio, ref: reference, Bold: statistically significant with $p<0.05$
Factor 1 represents Individual and collective responsibility on health, Factor 2 represents trust in vaccination system and information, Factor 3 represents concerns for vaccine safety, Confidence intervals are noted in parentheses below the ORs

Model 1: unadjusted
Model 2: Adjusted for sex, age, education, comorbidity, marriage, job, living with children, income.
Model 3: Adjusted for sex, age, education, comorbidity, marriage, job, living with children, income, and Factor 1
Model 4: Adjusted for sex, age, education, comorbidity, marriage, job, living with children, income, and Factor 2
Model 5: Adjusted for sex, age, education, comorbidity, marriage, job, living with children, income, and Factor 3

Table S4. Multivariate Analysis for explanatory variables and Intention of COVID-19 vaccination

Explanatory Variables		Intention of COVID-19 vaccination			
		Crude OR	95%CI	Adjusted OR	95%CI
**Civic participation**	No participation	1.00	ref	1.00	ref
	Any participation	**1.53**	**1.34, 1.76**	1.14	0.99, 1.32
**Social cohesion**	No social cohesion	1.00	ref	1.00	ref
	Any social cohesion	**2.07**	**1.86, 2.30**	**1.75**	**1.56, 1.95**
**Reciprocity**	No reciprocity	1.00	ref	1.00	ref
	Any reciprocity	**1.90**	**1.61, 2.24**	**1.42**	**1.18, 1.71**
**Sex**	Men	1.00	ref	1.00	ref
	Women	**0.86**	**0.77, 0.95**	0.91	0.81, 1.03
**Age**	20-59 years old	1.00	ref	1.00	ref
	60-69 years old	**1.86**	**1.65, 2.09**	1.30	0.81, 1.03
**Educational level**	Below university graduate	1.00	ref	1.00	ref
	University graduate or above	**1.40**	**1.26, 1.55**	**1.33**	**1.19, 1.49**
**Underlying disease**	None	1.00	ref	1.00	ref
	1	**1.65**	**1.44, 1.88**	**1.38**	**1.19, 1.58**
	2 or more	**2.42**	**1.96, 2.99**	**1.92**	**1.54, 2.40**
**Marriage Status**	Unmarried	1.00	ref	1.00	ref
	Married	**0.73**	**0.65, 0.81**	**0.86**	**0.76, 0.98**
**Job employment**	Unemployed	1.00	ref	1.00	ref
	Employed	**0.78**	**0.66, 0.91**	1.02	0.85, 1.22
**Living with children under 12 years old**	No	1.00	ref	1.00	ref
	Yes	**0.79**	**0.69, 0.91**	**0.81**	**0.69, 0.95**
**Household Income***	-4,000,000	1.00	ref	1.00	ref
	4,000,000-8,000,000	1.08	0.95, 1.24	1.11	0.95, 1.29
	8,000,000-	1.18	0.99, 1.39	1.11	0.92, 1.33

OR: odds ratio, ref: reference, Bold: statistically significant with $p<0.05$
*Household Income in Yen
